# “Small Blood Vessels: Big Health Problems?”: Scientific Recommendations of the National Institutes of Health Workshop

**DOI:** 10.1161/JAHA.116.004389

**Published:** 2016-11-04

**Authors:** Francesca Bosetti, Zorina S. Galis, Margaret S. Bynoe, Marc Charette, Marilyn J. Cipolla, Gregory J. del Zoppo, Douglas Gould, Thomas S. Hatsukami, Teresa L. Z. Jones, James I. Koenig, Gerard A. Lutty, Christine Maric‐Bilkan, Troy Stevens, H. Eser Tolunay, Walter Koroshetz, the “Small Blood Vessels: Big Health Problems” Workshop Participants, Dritan Agalliu, David A. Antonetti, Manfred Boehm, Claudette E. Brooks, Kathleen M. Caron, William Chilian, Mat J. Daemen, Robert D'Amato, Thomas P. Davis, Adviye Ergul, James E. Faber, Ariel R. Gomez, Peter Grayson, Isabella Grumbach, Jaime Grutzendler, Chenghua Gu, David Gutterman, John Hallenbeck, Ira Herman, Jay Humphrey, Costantino Iadecola, Edward W. Inscho, David Kleinfeld, Eng H. Lo, Jose A. Lopez, Stephen Macknik, Asrar Malik, Tanya N. Mayadas, Dorian McGavern, Gerald A. Meininger, Virginia M. Miller, Maiken Nedergaard, Mark T. Nelson, Shayn Peirce‐Cottler, Ipolia Ramadan, Gary A. Rosenberg, Ernesto L. Schiffrin, Peter Searson, Nina Stachenfeld, Radu V. Stan, Yajaira Suarez, Eroboghene E. Ubogu, Zinaida S. Vexler, Cornelia M. Weyand, Berislav V. Zlokovic

**Affiliations:** ^1^ National Institute of Neurological Disorders and StrokeNational Institutes of Health (NIH)BethesdaMD; ^2^ National Institute of Diabetes and Digestive and Kidney DiseasesNational Institutes of Health (NIH)BethesdaMD; ^3^ National Heart, Lung and Blood InstituteNational Institutes of Health (NIH)BethesdaMD; ^4^ Cornell UniversityIthacaNY; ^5^ University of VermontBurlingtonVT; ^6^ University of WashingtonSeattleWA; ^7^ University of CaliforniaSan FranciscoCA; ^8^ Johns Hopkins UniversityBaltimoreMD; ^9^ University of South AlabamaMobileAL

**Keywords:** endothelium, hypertension, imaging, ischemia, microcirculation, remodeling, Biomarkers, Endothelium/Vascular Type/Nitric Oxide, Hemodynamics, Inflammation, Vascular Biology, Pathophysiology, Remodeling, Hypertension, Blood-Brain Barrier, Cognitive Impairment

## Introduction

Small blood vessels (generally <100 μm in internal diameter) contribute to fundamental physiological processes and pathological events, but may not necessarily garner the attention associated with macrovascular physiology and disease. Major reasons for the bench‐to‐bedside research gap is the complexity and the size of small vessels throughout the body. Small vessels contain diverse cellular components and interact with a large variety of nonvascular parenchymal cell populations that differ among various organs. Depending on their location, the overlapping effect of environmental, epigenetic, and developmental factors adds to this complexity, challenging the translation of fundamental discoveries to the bedside. A better understanding of the specific structural and functional signatures of small vessels throughout the body and how their local perturbations can contribute to systemic pathophysiological conditions has the potential to transform diagnostic and therapeutic approaches.

To advance this important area of science, the National Institutes of Health held a workshop on September 18–19, 2014 that brought together scientists and clinicians from diverse areas of microvascular research to share their latest discoveries, identify common challenges, and foster collaborative research on the physiology and pathology of small blood vessels in many organs and tissues. The workshop included 7 scientific sessions, entitled: (1) Basic Biology and Natural History of Small Vessels; (2) Vascular Dynamics; (3) Small Vessel Cellular Interactions; (4) Transendothelial Transport, Including Across the Blood Brain Barrier (BBB) in Health and Disease; (5) Small Vessels in Disease; (6) Effects of Internal Milieu and Disease on Small Vessels; and (7) Research Tools and Innovation. All sessions and panel discussions are available in the National Institutes of Health Videocast archive (http://videocast.nih.gov/PastEvents.asp). This white paper is not meant to be a comprehensive review of the topics. Rather it is meant to articulate the gaps and opportunities identified by the workshop participants. We regret any major omissions that might have occurred. The top scientific priorities identified by participants needing further study are summarized in Table.

**Table 1:** Top Scientific Priorities From the National Institutes of Health (NIH) Workshop “Small Blood Vessels: Big Health Problems?”

Basic biology and natural history of small vessels	Understand the mechanisms driving complex local specialization of endothelial cells and development of small vessels, in order to identify therapeutic targets that take into account the heterogeneity in structure and function of the endothelium between distinct organs and within a tissue and the influence of genetic determinants, sex, hormonal status, and age
Vascular dynamics	Visualize with spatial and temporal fidelity the critical subcellular signal transduction networks, intermolecular interactions (eg, molecular anatomy), and cell–cell and cell–matrix properties in health and disease
Small vessel cellular interactions	Understand the molecular and cellular processes in homeostasis and response to injuries of small vessels, and how cellular and organ‐specific environments influence this response
Transendothelial transport, including blood–brain barrier, in health and disease	Deconstruct the regulation and function of the neurovascular unit (including adhesion, extracellular matrix, tight and adherens junctions and transcytosis) in health, and reconstruct them in disease
Small vessels in disease	Develop translational, mechanism‐based therapies to prevent or slow progression of small vessel diseases and define which patients to treat, and when and how to treat them
Effects of internal milieu and disease on small vessels	Develop better and clinically relevant models of diseases of small vessels and elucidate the interactions between vasculature, inflammation, and immune activation across the lifespan
Research tools and innovation	Develop and integrate synergistic biological, technological, and computational advances in order to understand complex, dynamic interactions among different signaling pathways, cell types, cells and matrix proteins, small and large vessels, and vessels and their microenvironments, through multidisciplinary teams

## Basic Biology and Natural History of Small Vessels

The common structural component of all small blood and lymphatic vessels throughout the body is the endothelium. The endothelial layer is the only common cellular component of capillaries, the simplest vascular structures with the smallest diameter. While vascular smooth muscle cells surround the endothelial layer in arterioles and venules, outside the brain pericytes are quite abundant on small venules and arterioles but are rather sparse on capillaries.^1^ Within the brain, however, there is controversy about pericyte coverage of capillaries.^2^ Heterogeneity in endothelial cells is particularly evident at the level of capillaries, where endothelial cells specifically adapt to the needs of the surrounding tissues.^3^ The endothelium varies in structural appearance in different organs and may be continuous, discontinuous, fenestrated, or sinusoidal in nature. Beyond controlling the highly specialized blood–tissue exchanges needed for nutrient transfer, signaling, or immune function, the endothelium performs other multiple key physiological functions, including maintaining antithrombotic surface and in arterioles the vasomotor tone.^4^ In fact, the endothelium can be viewed as one organ, a giant mosaic whose parts follow patterns that are shaped by environmental and developmental factors.^5^, ^6^

**Figure 1:**
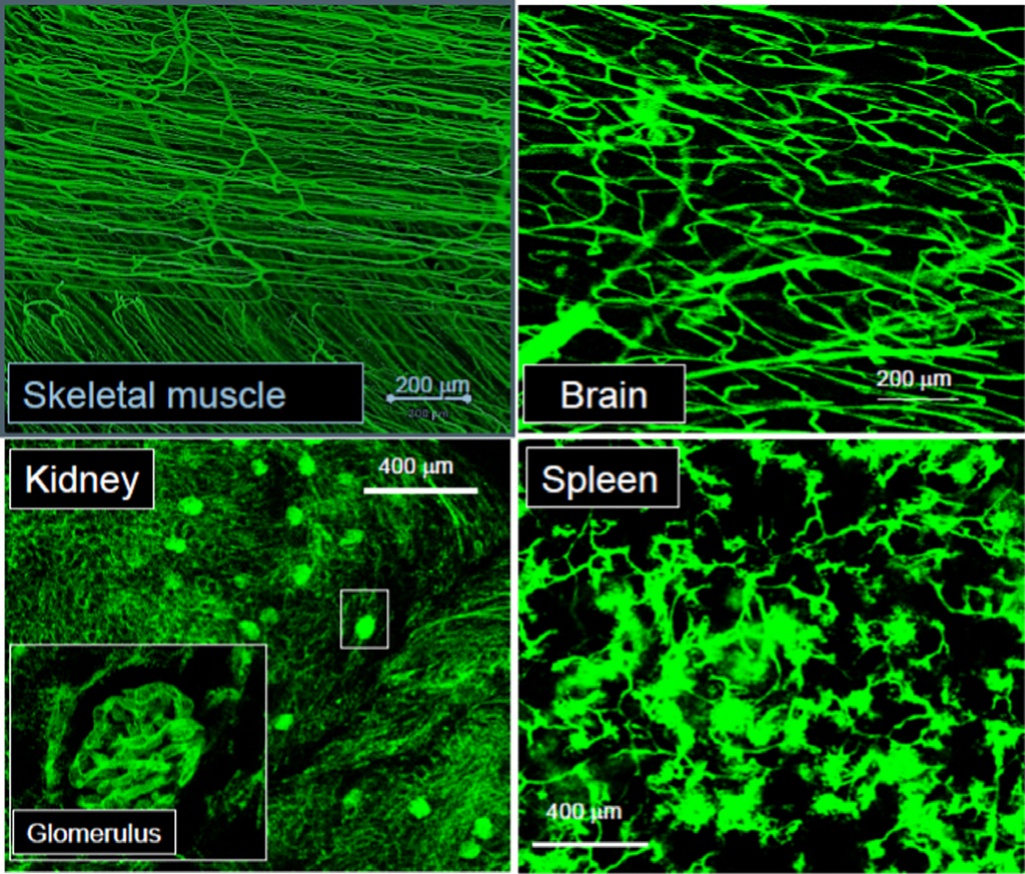
Differences in the functional small blood vessel architecture and normal perfusion of various mouse organs. Systemically injected fluorescent microspheres are tightly contained with the vascular structures with continuous endothelium, exemplified in skeletal muscle and brain by the smooth appearance of the small vessels. In contrast, microspheres cross through the fenestrated endothelium of kidney glomeruli and escape through the pores of the discontinuous endothelium of spleen sinusoids (Zorina Galis, unpublished data).

Much remains to be learned about the mechanisms involved in vascular development, including the bridge that connects vascular development with endothelium specialization; the molecular and physical factors that determine the precise temporal arrangement, shape, branching, and size of the vasculature; and the elements that modulate endothelial barrier function (Figure 1).^7^ This session identified the need for interdisciplinary teams to help decode the entire normal and pathological tissue‐specific molecular heterogeneity patterns of the endothelium. This knowledge is needed to develop better prognostic and diagnostic markers for a variety of local and systemic diseases, and might one day allow the precise tissue targeting of new therapies by delivery to the correct “endothelial ZIP‐code.”^8^

The function of the endothelial lining involves significant “cross‐talk” within the vessel wall, between the endothelial and mural vascular cells, and between bloodborne cells and signals, small vessels, and their surrounding tissues. Integration of the vascular structure and function across spatial and temporal scales, from genes to tissue, is key to understanding vascular development and physiological and pathological remodeling. Angiogenesis, arteriogenesis, the growth of collateral vessels, and the regression of vascular structures are dynamic events that can occur over a relatively short time span. These remodeling phenomena may affect an individual's propensity to adverse events such as myocardial infarction and stroke, and their manifest severity, and are influenced by individual genetic and environmental factors.^9^, ^10^ Different strains of mice exhibit differences in arteriogenesis, depending on the location of preexisting connections between arterioles, as well as differences in their susceptibility to disease,^11^ supporting the role of genetics in vascular remodeling. For instance, the density of brain collateral circulation is strongly determined by genetic background and is an important determinant of stroke outcome in animals and potentially humans.^12^, ^13^

Similarly, facets of the lymphatic system that can influence inflammation and the vascular response to disease are also genetically determined.^14^ It is less clear, however, what particular genes specify variation in the formation, maintenance, and remodeling of small vessels. Furthermore, there is a paucity of information about how genetic variants influence the response of small vessels across the lifespan. Some genetic susceptibility factors seem to be shared across different manifestations of small vessel disease.^15^

Recent data indicate that pericytes can control microvascular remodeling and angiogenic switching.^16^ “Angiophagy,” leading to the loss of vascular structures (also recognized in aging), also emerged as potentially critical for vascular occlusion and recanalization.^17^, ^18^, ^19^ It is recognized by this session that new computational approaches that could integrate all factors that affect vascular development and remodeling to simulate these processes using stochastic or deterministic rules could greatly advance the ability to predict individual risk and possibly prevent disease.

Sex is a critical biological determinant that modulates microvascular pathophysiology.^20^ The relative concentration of sex hormones that exist both in males and females varies, which means the sex biological biasing factors vary across the lifespan.^21^ Sex differences occur in incidence and presentation of cardiovascular disease, chronic kidney disease, and vascular dementia.^22^, ^23^, ^24^ Preeclampsia, menopause, and erectile dysfunction are sex‐specific conditions reflexing the complement of sex chromosomes and sex hormones,^25^ and are associated with small blood vessel dysfunction, but we still do not understand these associations at a fundamental level. Hormonal variation affects expression of genes, and mosaicism of X inactivation in females may not be as random as previously thought. Interestingly, changes in small vessel structure (such as remodeling) and function can be markedly different in males and females, or in response to sex hormones. For example, fluctuating levels of sex hormones have important consequences on stroke outcome in female animals, and marked changes in the endothelial lining of small blood vessels appear during pathologies associated with pregnancy such as preeclampsia and eclampsia.^26^, ^27^, ^28^, ^29^, ^30^ In the peripheral microcirculation, endothelin‐mediated vasoconstriction or nitric oxide–mediated endothelium‐dependent vasodilation is significantly affected by sex steroids and may underlie pathology in polycystic ovary syndrome.^31^ Therefore, careful attention should be given to sex and reproductive history in experimental design for both preclinical and clinical research. An integrative approach is needed to discover how sex affects vascular function within all components of the circulatory system.

To advance the understanding of small vessel disease, cross‐disciplinary research and the development of new tools that include computational modeling and imaging could enable integration of data across scales, ranging from molecular to tissue levels. Additionally, the field needs to better understand the mechanisms driving complex local structural and functional specialization of endothelial cells and how blood vessels develop and remodel across the lifespan. A better definition of “small vessel disease” that can affect the brain, heart, kidney, or other organs, as well as an integrative approach to discerning causation of small vessel disease are needed to identify new diagnostics and therapeutic targets and biomarkers.

## Vascular Dynamics

The vascular tone (resistance) is regulated to match blood flow to metabolic demand in order to provide adequate profusion to tissues under different physiological conditions. As an example, afferent and efferent arteriole resistances are differentially controlled to regulate filtration dynamics in the glomerular capillaries that lie in between these portal resistance elements. Mechanical forces are stimuli that mediate these regulatory effects. Mechanosensation of blood pressure and flow contributes to vasculogenesis and vascular remodeling. Alterations in transduction of the mechanical signal, ie, mechanotransduction, are associated with disease processes such as hypertension, diabetes, atherosclerosis, and large vessel stiffening. Although many putative mechanotransducers have been identified,^32^ much work remains to be done to understand in detail how these various components orchestrate responses to shear stress.

Many challenges remain for understanding vascular dynamics. At the macro‐scale, it is important to identify specific sensors in endothelial and vascular smooth muscle cells, their biochemical and cellular signaling pathways in different organs, and their synergy with systemic risk factors in disease progression. At the meso‐scale, work is needed to characterize microvascular cellular topology in relationship to organ function. At the micro‐scale, it is important to elucidate how single cell function within small blood vessels themselves influences the vasculature. These goals can be achieved through a better understanding of microanatomy and microphysiology, genomics, proteomics, and metabolomics throughout the circulatory system. Opportunities for scientific advances include elucidating the heterogeneity of cellular phenotypes; understanding the mechanics and development of the matrix, and the long‐range interactions (mechanical, electrochemical, and bloodborne); developing cell‐free scaffolds to be used for tissue engineering; imaging in 3 dimensions with high temporal and spatial resolution; and mathematical modeling to integrate these concepts with real vascular dimensions, functions, and biochemical processes.

Several gaps remain in our understanding of the role of the microcirculation in important fundamental mechanisms in health and in disease, such as metabolic hyperemia and the etiology of small vessel disease (SVD). For example, much remains to be understood regarding the causal relationship between coronary microvascular disease and ischemic heart disease. New methods for high‐resolution *ex vivo* imaging and morphometry of the entire coronary vasculature in pigs, other large mammals,^33^and mice^34^ will help fill these gaps. However, technological advances, such as biosensors for understanding intracellular and macro‐scale signaling and mechanics; higher resolution and speed, imaging technologies that can penetrate into deeper layers of tissues in vivo, including intrinsic fluorescence; fate mapping for disease etiology, eg, site for angiogenesis; and new transgenic and conditional animal models are needed to further advance the field.

## Small Blood Vessel Cellular Interactions

Although the microvasculature shares common structural and functional characteristics in many organs, there are also important organ‐specific features. Endothelial cells of small vessels closely interact with smooth muscle cells, pericytes, and tissue‐specific nonvascular cells, as well as circulating or tissue resident immune cells. Vascular smooth muscle cells interact and communicate with endothelial cells in the control of vascular tone. Nonvascular and vascular interactions shape the physiological responses and pathophysiological outcomes following injury and in microvascular complications.^7^ These interactions are in turn influenced by microenvironment and organ‐specific drivers.^35^ For instance, antibody‐mediated inflammation and injury of glomerular capillaries, which carry out the first step of filtering blood, underlie many glomerular disorders in the kidney.^36^

The immune system changes with aging toward a more aggressively pro‐inflammatory state. Alterations in the adaptive and innate immune system contribute to age‐associated morbidity and mortality, and increased pro‐inflammatory cytokines (eg, interleukin‐6, tumor necrosis factor‐α) in aging have been associated with hypertension, atherosclerosis, dementia, and diabetes, among other diseases. However, there is a strict territorial behavior of inflammatory disease, and aging is not always the cause. Elucidating the role of inflammation on vascular health and disease constitutes an important scientific gap.

Many features of the immune interactions with small blood vessels remain poorly understood. These immune interactions include target organ susceptibility factors that dictate the extent of the immune response and damage initiated by deposited antibodies^37^; differences in the characteristics of immune responses following deposition of soluble immune complex versus in situ immune complex formation; and the mechanisms by which neutrophils and their cross‐talk with other immune cells induce injury. For example, in the kidney, work is needed to understand how immune cells contribute to chronic glomerulonephritis; how glomerular inflammation leads to tubulo‐interstitial damage, which predicts the progression to end‐stage renal disease; and, how microvascular inflammation increases the risk for macrovascular disease (eg, atherosclerosis). Some similarities between the pathology of small blood vessel disease in the kidney and the brain suggest that similar mechanisms may be at work.

Excessive oxidative stress of cells of the vascular wall resulting from altered metabolism, inflammatory cytokines, or mechanical forces contributes to vascular diseases. However, large clinical trials in which antioxidants have been given in high‐risk patients for the prevention of cardiovascular events did not demonstrate the expected benefits,^38^, ^39^, ^40^ in part because redox‐responsive pathways that induce specific organ pathology have remained elusive. Obstacles to deciphering specific redox‐dependent pathways are the localized nature of reactive oxygen species (ROS) production (eg, in caveolae, lipid rafts, endosomes, or mitochondria), the particular properties of the oxidants (eg, superoxide, peroxynitrite), and the plethora of chemical protein modifications (eg, sulfenylation, glutathionylation) and their reversible nature.^41^ New technologies to resolve intracellular reactive oxygen species spatially and temporally, integrated in silico algorithms to predict redox modifications, and reliable methods to identify redox modifications by mass spectroscopy would facilitate progress in understanding vascular oxidative stress. Additionally, redox signaling occurs in parallel to other key signaling events, eg, calcium‐ and reactive oxygen species–dependent signaling pathways, which can interact and modulate the activity of each other.^42^ An integrated approach is required to determine the physiological relevance of specific vascular redox pathways and their interplay with other key processes in the adjacent cells and within tissues.

*Ex vivo* vascular modeling systems could provide the opportunity to study disease mechanisms. Such systems have been used to investigate thrombosis, in particular, self‐assembly of von Willebrand factor, endothelial barrier function, angiogenesis, and diseases affecting small vessels including thrombotic microangiopathies (including thrombotic thrombocytopenic purpura), sepsis, malaria, and sickle cell disease.^43^ These systems allow the effect of changes in different physiological or pathological parameters, such as shear stress, flow rates, vessel geometry, and role of other cell types to be studied in real‐time. However, the systems need to be improved to overcome low throughput and developed to apply to the central nervous system (CNS) and other organs.

## Transendothelial Transport, Including Across the BBB, in Health and Disease

**Figure 2:**
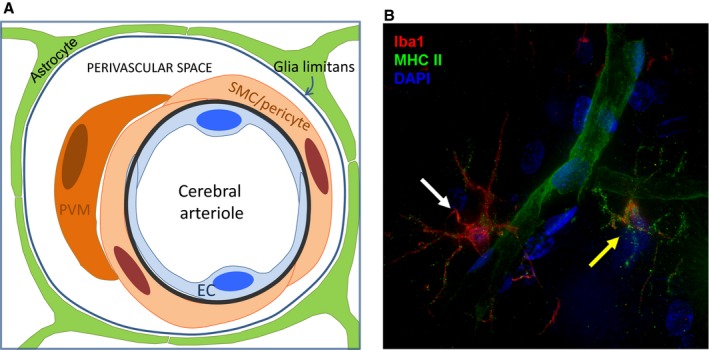
A, The neurovascular unit at the level of a pial arteriole (Courtesy of Dr Giuseppe Faraco, Weill Cornell Medical College). B, Perivascular microglial cells. This deconvolution fluorescence microscopy image illustrates the proximity of microglial cells to a cerebral capillary in the adult rat hindbrain. A 30‐μm frozen rat brain section was stained for Iba1 (red), a microglial marker, and MHC II (green), which is upregulated in activated microglia but also stains endothelial cells. Nuclei were stained with 4′,6‐diamidino‐2‐phenylindole (DAPI) (blue). Microglia are the resident macrophage of the CNS and serve a number of roles including defense against pathogens that cross the BBB. Note the difference between the “surveillance state” microglia (white arrow), which has a small amount of punctate green MHCII staining on the processes and the “activated” microglia (yellow arrow), which has increased punctate MHCII staining that defines the outline of the processes. The capillary, indicated by the green MHCII staining, winds between the 2 microglial cells. This image is a maximum intensity projection of a 10‐μm‐thick segment of the brain slice. BBB indicates blood–brain barrier; CNS, central nervous system; EC, endothelial cell; PVM, perivascular macrophage; SMC, smooth muscle cell.

**Figure 3:**
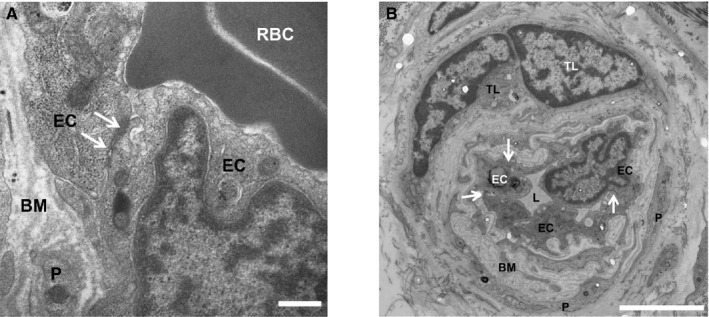
A, Tight junctions at the human blood–nerve barrier (BNB). A digital electron micrograph of the BNB in the sural nerve from an untreated adult patient with Guillain‐Barré syndrome shows intact electron‐dense intercellular tight junctions (white arrows). Scale bar=0.5 μm. B, Human BNB alterations in disease. A digital electron micrograph of the BNB in the sural nerve from an adult patient with chronic inflammatory demyelinating polyneuropathy shows BM thickening/duplication between endoneurial EC and pericytes (P). Intact electron‐dense intercellular tight junctions (white arrows) are seen. Perivascular T‐lymphocytes (TL), a common feature in immune‐mediated polyneuropathies, are also observed. Scale bar=5 μm. BM indicates basement membrane; EC, endothelial cell; L, lumen; P, pericyte; RBC, red blood cell.

The major microvascular barriers of the brain, the blood–brain barrier (BBB) and the blood–cerebrospinal fluid barrier, and of peripheral nerves, the blood–nerve barrier (BNB), share unique properties required to tightly regulate the transport of ions, solutes, nutrients, and macromolecules between the peripheral circulation and the neural parenchyma, as well as control hematogenous leukocyte trafficking during physiologic and pathophysiologic conditions. The BBB is a very restrictive layer of highly specialized endothelial cells that exist throughout the brain vessels, and is even more specialized in capillaries and the inner layers of postcapillary venules in the brain (Figure 2A). In the brain, processes from neighboring astrocytes and microglial cells contribute to the BBB, forming the neurovascular unit (Figure 2B). These cells can regulate barrier function, contributing to the internal homeostasis of the brain as required for signal transduction. Blood cells, particularly polymorphonuclear cells, lymphocytes, and monocytes, also interact with the endothelium and may affect the neurovascular unit.^44^ Similarly, the BNB is formed by specialized endothelial cells that form microvessels within the innermost layer of peripheral nerves and nerve roots (the endoneurium).^45^ These endothelial cells exhibit highly restricted pinocytosis and transcytosis potential, express specific transporters that regulate the influx/efflux of nutritive/toxic compounds, express low levels of leukocyte adhesion molecules under normal physiologic states and an array of specialized proteins that form tight and adherens intercellular junctions that efficiently restrict passive diffusion of bloodborne molecules and contribute to a high transendothelial electrical resistance (Figure 3A). In addition, extracellular matrix adhesion of endothelial cells by integrins can determine tight junction fidelity.^46^ These specialized endothelial cells are surrounded by pericytes that share a common basement membrane.

Altered BBB permeability has been implicated in small vessel disease, lacunar stroke, vascular dementia, and Alzheimer's disease.^47^ Alternations in BNB structure, such as basement membrane thickening (Figure 3B), have been described in chronic peripheral nerve disorders such as diabetic neuropathy and chronic inflammatory demyelinating polyneuropathy.^48^

A special paravascular space described in the rodent brain serves as a lymphatic system in the CNS.^49^ This highly polarized macroscopic fluid transport system has been termed the “glymphatic” system. Aging and microinfarcts appear to be linked to a marked reduction in glymphatic activity. Alzheimer's disease is also thought to be a consequence of altered perivascular drainage due to faulty removal of β‐amyloid from the brain by the glymphatic transport and accumulation of β‐amyloid proteins. The effects of changes in BBB permeability, hypertension, diabetes, SVD, and other comorbidities on glymphatic clearance, as well as the role of individual cell types in the neurovascular unit on glymphatic activity, are unknown. More importantly, the presence of the glymphatic system in the human brain remains to be demonstrated, which requires development of human biomarkers of glymphatic activity.

Cerebral SVD can be sporadic or hereditary, and is characterized by leakage of plasma proteins into the vessel walls and perivascular spaces. SVD is thought to play a role in the pathogenesis of neurodegenerative diseases including Alzheimer's disease, dementia, and cognitive decline, retinal vasculopathy with cerebral leukodystrophy, white matter disease, and lacunes of the central gray matter. The processes initiating SVD of the CNS are not exactly known but may involve hypoxia/ischemia, inflammation, pericyte degeneration, and alteration in BBB capillary function, resulting in BBB leakage. Improvements in neuroimaging and studies in animal models are needed to address gaps in knowledge of the etiology, risk factors, or triggers of SVD.

Transcellular transport is another dynamic process that needs to be further elucidated. Depending on the tissue or the disease state, the passage of a macromolecule from peripheral circulation into the CNS or peripheral nerves could be the result of increased paracellular entry attributable to alterations in intercellular tight junction function or an active transport process (eg, receptor‐ or caveolae‐mediated transcytosis). Advancing knowledge of the repertoire of transporters and their function, expression, and regulation would help improve small molecule drug delivery into the brain and peripheral nerves, and in turn, treatment of neurological and psychiatric disorders.

Additional gaps in knowledge and areas for future research include a better understanding of junctional formation and disassembly of the BBB and BNB, such as signaling pathways that reassemble both adherens and tight junctions to restore endothelial barrier; the precise workings of endothelial transcytosis pathways and transcellular pores and means of exploiting them to deliver polar or large drugs across restrictive barriers; mechanisms by which the endothelial barrier coordinates the passage of fluid and solutes as well as circulating hematogenous and neoplastic cells; reasons underlying the incapacity of some microvascular barrier systems to adequately repair; and the restoration of endothelial integrity. We also need to identify differences among developmental/reparative angiogenesis, collaterogenesis, and pathological neovascularization, develop therapeutic interventions to promote vascular repair, and better understand the role and interactions of cellular and humoral factors in microvascular barrier formation and adaptation, in health and disease.

## Small Vessels in Disease

Microvascular complications are well‐recognized effects of longstanding hypertension or diabetes.^50^ In addition, small blood vessels themselves cause or contribute to a diversity of diseases and pathogenic processes, such as hypertension, coronary microvascular disease, pulmonary hypertension, sepsis, focal strokes, microinfarcts, large cerebral hemorrhages or microscopic hemorrhages (microbleeds), and dementia.^26^ Additionally, subtle and chronic changes in vascular structure and function can lead to cerebral white matter damage and cognitive dysfunction. These are accompanied by increased BBB permeability, endothelial dysfunction, and alterations in myogenic tone, neurovascular coupling, and consequently cerebrovascular autoregulation.^27^ Hypertension is a risk factor for Alzheimer's disease and increases in blood pressure are linearly related to a decrease in cognitive abilities.^28^ Deleterious microvessel changes, such as those underlying chronic hypertension, can occur very early and exacerbate other pathological processes in many tissues and organs.^51^ For instance, diabetes superimposed on hypertension exacerbates small artery remodeling and the degree of remodeling is greater in diabetics than in nondiabetic hypertensive patients.^52^, ^53^ In the kidney, glomerular injury leading to renal insufficiency can arise from hypertension, impairment of microvascular function, inflammatory insult, or autoimmune conditions.^36^, ^54^ Over prolonged periods, the impairment in vascular regulation in the kidney could accelerate progression of renal injury, and in the brain it could lead to cognitive impairment via chronic mismatch between the delivery of nutrients through blood flow and the brain high energy requirements, particularly in the subcortical white matter.^24^

Large cerebral arteries, pial arterioles, intraparenchymal arterioles, capillaries, and venules react in a dramatically different way to hypertension. Stiffening, atherosclerosis, remodeling, collagenosis, and lipohyalinosis are observed in different vascular districts, resulting in distinct pathologies. For example, in the presence of atherosclerosis, the coronary endothelial vasomotor response to acetylcholine is frequently reduced but is variable between patients,^55^ and in the CNS large vessel atherosclerosis may lead to vascular occlusion and focal stroke, small vessel atherosclerosis to microinfarcts, lipohyalinosis to white matter disease, and venous collagenosis may promote hemorrhage or microbleeds.^26^ However, it remains to be established how different vascular segments of the cerebral microcirculation react to, or contribute to the etiology of essential hypertension and what the underlying mechanisms are. Therefore, it is important to investigate the effect of hypertension and other risk factors on different segments of the circulation and identify relevant cellular and molecular mechanisms leading to vascular dysfunction and damage, particularly at the level of small vessels.

One major challenge that lies ahead is to develop translational, mechanism‐based therapies to prevent, or slow, progression of small vessel disorders. The key components of this intervention are defining who, when, and how to treat. Accurate assessment of vascular health is complicated because microvessels are small, and within the tissue parenchyma, relatively inaccessible to observation without invasive biopsy, with few exceptions (eg, retina).

Finding treatments for vascular cognitive impairment has been impeded by patient heterogeneity and lack of biomarkers of key microvascular processes related to cognitive and neurologic impairment. Identifying homogeneous subgroups of vascular cognitive impairment patients (eg, Binswanger's disease) by multimodal biomarkers‐based clinical examination, magnetic resonance imaging studies of BBB permeability and microstructure, and cerebrospinal fluid studies indicative of inflammation, which indicates pathophysiology of white matter injury, would accelerate development of effective treatments.^56^ Still needed are validations of a biomarker approach in larger populations, identification of outcome measures, and development of drugs to test in treatment trials.

Achieving rapid, robust, and noninvasive assessment of vascular health with sufficient resolution is a major challenge, especially for microvessels. Technological advances to develop noninvasive monitoring and improve imaging techniques to longitudinally visualize small vessels without biopsy will allow early detection and monitoring of disease progression and provide a greater appreciation for the natural history of SVDs.

Clinical and preclinical studies to identify sensitive and accurate circulating (and nonvascular) biomarkers as surrogate readouts of vascular health will represent critical advances. Development of methods for rapid, robust, and noninvasive sampling of vascular health will allow early detection, early intervention, and better assessment of disease progression in patients. Earlier detection may lead to identification of underlying contributing factors, including potentially modifiable risk factors that do not require therapeutic interventions. Identifying the earliest signs of disease by advanced imaging or circulating biomarkers will also define a therapeutic window, and guide the selection of patients to treat and the time of treatment. The ability to noninvasively monitor disease progression, stability, or regression can inform modifications to patient care. Importantly, accurate and robust methods to quantify these parameters will improve the ability to critically assess early success or failure of potential therapies in clinical trials. As methods for advanced detection become easier, faster, and cheaper, they may be adopted as accepted surveys of vascular health, resulting in a greater number of patients being tested. This will translate into more powerful disease‐prevention efforts and improved vascular health.

Genetic investigations will continue to be important in defining, identifying, and treating those patients who are at greatest risk of vascular disease. With increasing cohort sizes in highly powered genomewide‐association studies that are dichotomized by sex and the increasing availability of personalized data through genome or exome sequencing, the breadth and depth of understanding of genetic risk factors are increasing at unprecedented rates. The availability of individual genetic data will refine both general and personal genetic risk factors, which could be used to prioritize patients who should be closely monitored. For example, polymorphisms in novel genes that are responsible for variation in the extent of the native collateral circulation in brain and other tissues, as well as collateral remodeling in models of occlusive disease, recently have been identified in mice and are being investigated for similar impact in humans.^12^, ^57^, ^58^ Early identification and intervention for individuals at greatest genetic risk can allow prevention of microvascular diseases, or tributary insults, rather than treating patients long after disease has begun. Complex, multifactorial diseases are unlikely to have “magic bullet” therapies and will instead require treatment of mechanistically related subsets of patients. Greater definition of the genetic landscape and architecture for vascular disease will allow identification of individual signatures to genetically stratify clinical drug trials, which may avoid unnecessarily rejecting drugs that may be efficacious in subsets of patients or to better predict individual drug responses.

A critical step is the development and use of disease‐relevant animal models to understand the primary molecular mechanisms and to de‐convolute biological complexities arising from interactions between multiple processes (oxidative stress, inflammation, extracellular matrix) that can differ with sex, age, comorbidities, etc. The use of combinations of risk alleles and inbred strains of mice allows tight control and simplification of genetic factors that contribute to disease, making these complex interactions approachable. On the other hand, outbred animal strains and recently developed mouse genetic mapping populations are also useful to understand population responses and provide genetic diversity that may be more suitable for evaluating population results in preclinical models.^59^ Development and characterization of disease‐relevant animal models through hypothesis‐driven gene targeting or validations of human loci will be invaluable to understanding cellular pathways and preclinical testing of novel therapeutics. For example, CRISPR methodology has enabled construction of mouse lines with poor, intermediate, and abundant collaterals in brain and other tissue by mutation of a single base‐pair in an otherwise isogenic background, thus providing a model of variation in collateral circulation that is evident in humans.^57^ Collaborative investigations merging concepts from human studies with those from basic cellular and animal models are viewed as the best approach to hasten translation to improved patient care.

## Effects of Internal Milieu and Disease on Small Vessels

Small vessels respond to local signals and hemodynamic forces differently depending on their structure, location, microenvironmental and systemic milieu, and/or the presence or absence of disease. While prolonged changes in local internal environmental conditions, including hemodynamic, bloodborne, and local tissue‐derived stimuli, can lead to compensatory or pathological changes in the small vessels of the body, genetic contributions should also be considered.

It has become clear that small vessels are dynamic and can exhibit plasticity. Examples include pharmacological control of diabetes or hypertension, which can reduce damage to small vessels and pathologic formation of new vessels and restore normal function.^60^, ^61^

In diseases such as diabetes and stroke and in premature births, remodeling may include angiogenic responses resulting in newly formed vessels that may not be functional, and may serve only to exacerbate inflammatory processes within the body.^28^, ^30^ During hypertension, small vessel inward remodeling to generate smaller lumens and thicker walls can be protective of vascular injury by increasing vascular resistance and preventing high perfusion pressures from damaging downstream capillaries.^62^ However, smaller lumen diameters can produce chronic hypoperfusion and infarction, and can limit vasodilator reserve in an organ as well as increase total peripheral resistance, which contributes to the hypertension.^63^ While this response to hypertension has been known for decades, how it differs in males and females, in response to hormonal status, or the influence of genetics is not known. In addition, how small blood vessels respond differentially to disease in different organs is largely unknown. An important example is preeclampsia, which affects the small vessels of many organs including the kidney, placenta, brain, and heart.^10^ How the small vessels of each organ are affected during preeclampsia may be significantly different yet important to understand for effective treatment and prevention of this condition.

## Research Tools and Innovation

Complex interactions among cell types influence both the micro‐ and macroenvironments of the vasculature. Overlapping processes that may be deleterious in an acute phase of disease or injury may be adaptive and beneficial during healing and repair. Thus, it is important to consider that responses to disease and injury may be time‐ and cell‐type dependent, as well as organ specific. To study them, there is a continuing need for advances in technologies for systematic measurement, including imaging, genomics, proteomics, bioinformatics, and similarly in mathematical and computational models to describe and predict complex dynamic behaviors.^64^

Model systems have played an important role in elucidating mechanisms of pathology and disease, and in the development of therapeutic interventions. Recent advances in bioengineering, stem cell technology, and microscopy have led to emergence of model systems that will accelerate scientific discovery and translation.^65^ Techniques such as 2‐photon microscopy and optogenetics^2^ provide new insights into the physiology and pathology of vascular disease in animal models. In vitro models are being developed that utilize tissue engineering and stem cell technology to recapitulate the vascular microenvironment of different organs with human cell lines. Computational models have been important in understanding circulation at macroscopic length scales, and advances in computational power are enabling the development of models at the cellular and molecular level. These emerging models are capable of simulating membrane dynamics, receptor/ligand interactions, transcellular transport processes, and in the near future will be capable of simulating vessel growth and remodeling.^66^

Currently, heterogeneous groups of disease processes are often lumped into single diagnoses (eg, vascular cognitive impairment or hypertension). Moreover, some disease processes are thought to be due primarily to large vessel disease, while others are attributed to small vessel disease. There is a strong need to better delineate the subtleties of disease and interactions among large and small vessels as well as among different vascular regions (eg, extracranial and intracranial, or aortic and renal).^67^

Integrative, multidisciplinary approaches are needed to improve tools for visualization and analysis suitable for understanding systems biology and pathophysiology across scales, in both animal models and individual patients, and for informing and validating much‐needed integrative, multiscale computational models. For example, there is a need for better information on 3D structure, function, and hemodynamic conditions in small‐to‐large vessels in different, yet interconnected, vascular beds,^68^, ^69^, ^70^ as well as for better validation of both the integrity of the data and the reliability of the methods used to analyze or simulate them.

The field should leverage emerging tools to improve our basic understanding of microvascular function in health so that we can delineate alterations in disease, including the development of high‐throughput metabolomic screens; mapping epigenetic changes in health and disease; and a better understanding of the role of the microenvironment on microvessels in different tissues.

Emerging data suggest that the small vessel “vasculome,” defined as the integration of RNA expression, epigenetic, protein, and metabolomic profiles, is organ specific and may contribute to disease pathophysiology and biomarker generation.^71^ Mapping the functional “vasculome” in different organs, and having an integrated vasculome database for all major target organs could provide a unique resource/tool for pursuing therapeutic and diagnostic leads in disorders of the small vessels. Possible differences due to sex, race, and age, which could impact drug delivery, drug penetration, uptake, and therapeutic outcome, also remain largely understudied. Finally, the field needs to create better opportunities for the exchange of ideas and data, particularly across disciplines, from basic scientists and engineers to clinicians. To achieve this, precise terminology, standardization, and sharing of data acquisition and analysis technologies as well as interdisciplinary collaborations are needed.

## Summary and Future Directions

The workshop provided a first‐time opportunity for scientists from different disciplines to come together and identify common challenges and scientific opportunities for basic and clinical research on small blood and lymphatic vessels. Among the cross‐cutting priorities, participants identified the need to expand our knowledge of the basic biology of small vessels; the structural and functional heterogeneity and differential susceptibility to injury and disease; the interactions of vascular cells with their neighboring cells and circulating factors; the molecular mechanisms of physiological and pathological autoregulation of small vessels in different organs; and the tissue‐specific phenotypes of endothelial cells, pericytes, or vascular smooth muscle cells that contribute to different vessel dynamic responses. An appreciation of factors, such as sex, hormonal status, developmental stage, age, comorbidities, and genetics are critical for understanding the complexity of the physiological and pathological responses of small blood vessels. Novel integrated multidisciplinary approaches to address computational modeling, noninvasive imaging, genetic determinants, and development of new animal models are key to address the pathophysiology of small blood vessels.

A challenge of this 2‐day workshop was to formulate specific and translatable recommendations that would be broadly applicable across different organs, diseases, and disciplines. Future endeavors and targeted initiatives aimed at tackling specific problems in greater detail are warranted. In this regard, in addition to supporting interdisciplinary research through team science grants, (P01, RC2, R24 etc), database resources for –omics research, human tissue resources, and conference grant support (U13 and R13), the National Institutes of Health is  currently considering or has started initiatives targeting small vessels, for example: M2OVE Alzheimer's disease (AD) (https://www.nih.gov/news-events/news-releases/decoding-molecular-ties-between-vascular-disease-alzheimers); Small Vessel Contributions to Cognitive Impairment and Dementia (VCID) Biomarkers Consortium: Coordinating Center (RFA‐NS‐16‐019); Small Vessel Contributions to Cognitive Impairment and Dementia (VCID) Biomarkers Development Projects (RFA‐NS‐16‐020); and Mechanistic basis of diffuse white matter disease in vascular contributions to cognitive impairment and dementia (RFA‐NS‐16‐021), which were informed by this workshop and other relevant planning efforts.^72^, ^73^, ^74^

## Appendix

Workshop participants (in alphabetical order): Dritan Agalliu, PhD, Columbia University Medical Center; William Aird, MDH, Harvard University; David A. Antonetti, PhD, University of Michigan; Manfred Boehm, MD, National Heart, Lung and Blood Institute, National Institutes of Health (NIH); Claudette E. Brooks, MD, Office of Research on Women's Health, National Institutes of Health (NIH); Kathleen M. Caron, PhD, University of North Carolina at Chapel Hill; William Chilian, PhD, Northeast Ohio Medical University; Mat J. Daemen, MD, PhD, University of Amsterdam's Faculty of Medicine; Robert D'Amato, MD, PhD, Harvard University; Thomas P. Davis, PhD, University of Arizona College of Medicine; Adviye Ergul, MD, PhD, Georgia Regents University; James E. Faber, PhD, University of North Carolina at Chapel Hill; Ariel R. Gomez, MD, University of Virginia; Peter Grayson, MD, MSc, National Institute of Arthritis and Musculoskeletal and Skin Diseases, National Institutes of Health (NIH); Isabella Grumbach, MD, PhD, Iowa City VA Medical Center; Jaime Grutzendler, MD, Yale University; Chenghua Gu, PhD, Harvard University; David Gutterman, MD, Medical College of Wisconsin; John Hallenbeck, MD, National Institute of Neurological Disorders and Stroke, National Institutes of Health (NIH); Ira Herman, PhD, Tufts University; Jay Humphrey, PhD, Yale University; Costantino Iadecola, MD, Cornell University; Edward W. Inscho, PhD, University of Alabama at Birmingham; David Kleinfeld, PhD, University of California, San Diego; Eng H. Lo, PhD, Harvard University; Jose A. Lopez, MD, Puget Sound Blood Center, Seattle; Stephen Macknik, PhD, St. Joseph's Hospital and Medical Center, Arizona; Asrar Malik, PhD, University of Illinois at Chicago; Tanya N. Mayadas, PhD, Harvard University; Dorian McGavern, PhD, National Institute of Neurological Disorders and Stroke, National Institutes of Health (NIH); Gerald A. Meininger, PhD, University of Missouri; Virginia M. Miller, PhD, Mayo Clinic; Maiken Nedergaard, MD, PhD, University of Rochester; Mark T. Nelson, PhD, University of Vermont; Shayn Peirce‐Cottler, PhD, National Institute of Arthritis and Musculoskeletal and Skin Diseases, National Institutes of Health (NIH); Ipolia Ramadan, PhD, National Institute of Neurological Disorders and Stroke, National Institutes of Health (NIH); Gary A. Rosenberg, MD, University of New Mexico Health Sciences Center; Ernesto L. Schiffrin, MD, PhD, McGill University; Peter Searson, PhD, Johns Hopkins University; Nina Stachenfeld, PhD, Yale University; Radu V. Stan, MD, PhD, Dartmouth College; Yajaira Suarez, PhD, Yale University; Eroboghene E. Ubogu, MD, University of Alabama at Birmingham; Zinaida S. Vexler, PhD, University of California, San Francisco; Cornelia M. Weyand, MD, PhD, Stanford University; Berislav V. Zlokovic, MD, PhD, University of Southern California.

## Disclosures

Hatsukami: research grant from Philips Healthcare; Bosetti, Bynoe, Maric‐Bilkan, Charette, Cipolla, del Zoppo, Galis, Gould, Jones, Koenig, Lutty, Stevens, Tolunay, Koroshetz: None.
